# Vertical Dynamic Deflection Measurement in Concrete Beams with the Microsoft Kinect

**DOI:** 10.3390/s140203293

**Published:** 2014-02-19

**Authors:** Xiaojuan Qi, Derek Lichti, Mamdouh El-Badry, Jacky Chow, Kathleen Ang

**Affiliations:** 1 Department of Geomatics Engineering, University of Calgary, 2500 University Drive N.W., Calgary, Alberta T2N 1N4, Canada; E-Mails: xiqi@ucalgary.ca (X.Q.); jckchow@ucalgary.ca (J.C.); kdang@ucalgary.ca (K.A.); 2 Department of Civil Engineering, University of Calgary, 2500 University Drive N.W., Calgary, Alberta T2N 1N4, Canada; E-Mail: melbadry@ucalgary.ca

**Keywords:** Microsoft Kinect, quantization error, structural deflection measurement

## Abstract

The Microsoft Kinect is arguably the most popular RGB-D camera currently on the market, partially due to its low cost. It offers many advantages for the measurement of dynamic phenomena since it can directly measure three-dimensional coordinates of objects at video frame rate using a single sensor. This paper presents the results of an investigation into the development of a Microsoft Kinect-based system for measuring the deflection of reinforced concrete beams subjected to cyclic loads. New segmentation methods for object extraction from the Kinect's depth imagery and vertical displacement reconstruction algorithms have been developed and implemented to reconstruct the time-dependent displacement of concrete beams tested in laboratory conditions. The results demonstrate that the amplitude and frequency of the vertical displacements can be reconstructed with submillimetre and milliHz-level precision and accuracy, respectively.

## 1. Introduction

Bridge structures are a major component of the civil infrastructure of any country. Like any other structure, bridges are designed and built to be safe against failure and to perform satisfactorily over their service life. However, over the past few decades, bridge infrastructure in many parts of the world has been deteriorating at an alarming rate due to inadequate maintenance, excessive loading, economically driven design and construction practices and adverse environmental conditions. Therefore, structural health monitoring of such crucial infrastructure is important for ensuring both their safety and serviceability over their lifespan. Excessive deformations, particularly deflection under long-term effects [1,2] and repeated moving loads (e.g., due to traffic) is one of the major factors that can adversely affect the serviceability of a bridge structure. Throughout the entire life of a structure deflection must not exceed acceptable limits specified by the design codes and standards. Bazant *et al.* [3,4] compiled records of excessive deflection for a large number of concrete bridges in different parts of the world. In concrete structures, deflection increases with the reduction in stiffness when cracking of the concrete occurs. Cracking and deflection of concrete bridges can be controlled by providing appropriate amounts of pre-stressing reinforcement during construction [5]. However, when the serviceability of a concrete bridge is compromised by excessive cracking and deflection, a promising new technique to enhance performance and extend the service life of the bridge is to bond fibre- or steel-reinforced polymer sheets to the surfaces of the bridge elements. Prior to application of these sheets to actual bridges, their efficacy must be assessed through controlled laboratory testing in which the deflection of strengthened beam or girder specimens is measured under static monotonic and cyclic fatigue loading.

The accurate measurement of deflection of the laboratory specimens can be achieved with different sensors such as dial gauges, linear-variable differential transformers and laser displacement sensors (LDSs). All, however, suffer limitations: the collection of only one-dimensional data; limited measurement range; the high cost to deploy many sensors across an entire structure; and high potential for the sensors to be damaged during testing.

The effectiveness and high accuracy of remote optical measurement methods such as photogrammetry [6–12] and terrestrial laser scanning [13–15] have been demonstrated. Laser scanning systems are, however, best suited for the measurement of displacements under static loading conditions due to their sequential data capture. Though Detchev *et al.* [16] demonstrate a digital photogrammetric system for both static and dynamic load test measurement—their experiments were conducted concurrently as those reported herein—the cameras' low acquisition rate limits the loading frequency that can be measured to 1 Hz, whereas 3 Hz is normally required [17,18].

The relatively recent development of range camera technology has, however, opened the possibility of dynamic load measurements. Lichti *et al.* [19] reported submillimetre deflection measurement accuracy for concrete beams subjected to static load testing using a time-of-flight range camera. Qi *et al.* [20] reported submillimetre accuracy from their investigation into time-of-flight range camera measurements of concrete beams subjected to dynamic load testing performed at different loading frequencies.

The Microsoft Kinect is a triangulation-based range camera that has been used for many applications such hand gesture recognition [21–25] and detection of the human body [26]. The application of the Microsoft Kinect to structural measurement problems can be considered advantageous for several reasons. First, the Microsoft Kinect can acquire three-dimensional (3D) measurements of extended objects such as concrete beams at video frame rate (30 Hz). Second, in contrast to bulky laser scanner systems, it is a very compact sensor (∼30.5 cm × 7.6 cm × 6.4 cm and 1.4 kg) so it can be easily handled and deployed. Third, the cost of the Microsoft Kinect is extremely low (∼CAD 100) in comparison to terrestrial laser scanners (∼CAD 40,000+), time-of-flight range cameras (∼CAD 5,000) or even digital SLR cameras (∼CAD 400+). In considering these advantages, this paper reports on an investigation into the use of the Microsoft Kinect to measure the vertical deflection of a concrete beam subjected to cyclic loads in a laboratory, which simulates traffic loading on bridges.

The paper begins with a general overview of the Microsoft Kinect sensor in Sections 2 and 3 then describes the mathematical modeling for the measurement of the concrete beam deflection in response to cyclic loading. Section 4 presents the experiment design, data collection and the depth data segmentation and displacement signal reconstruction algorithms. Section 5 reports the results of measuring the deflection of a concrete beam under cyclic loading with the Kinect. These are followed by the conclusions in Section 6.

## 2. The Microsoft Kinect Sensor

**Figure 1.**
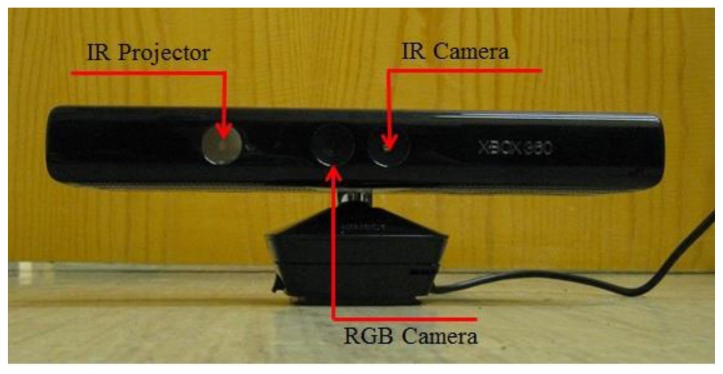
The Microsoft Kinect.

The Microsoft Kinect is based on PrimeSense chips and consists of an RGB camera, an infrared (IR) projector and an IR camera as illustrated in Figure 1. It is essentially a stereo vision system that determines depth by triangulation. The projector illuminates the scene with an infrared light speckle pattern generated from a set of diffraction gratings. The reflected speckle pattern is captured by the IR camera and is cross-correlated with a reference image. The reference image is obtained by capturing a plane at a known distance from the camera. The depth of a point in object space is determined by triangulation from the corresponding disparity between conjugate points [27].

The array size of the IR depth image is 640 × 480 pixels, which is smaller than the actual chip size (1,280 × 1,024 pixels) of the IR camera sensor, and has a pixel pitch of 5.2 μm and a 6.0 mm focal-length. The chip size is 6.66 × 5.33 mm^2^. The maximum frame rate of the Microsoft Kinect is 30 Hz. The depth measurement accuracy of the Microsoft Kinect degrades with increasing distances [28]. The lighting conditions can impact the computation of disparities; for example under strong sunlight, the laser speckles appear with low contrast in the infrared image.

The primary error sources include inadequate calibration and inaccurate disparity measurement. The different optical sensors of the Microsoft Kinect are affected by lens distortions (radial and decentring). In addition, the boresight and leverarms between the cameras may not be properly modeled. Such systematic errors can be reduced by a rigorous calibration procedure, e.g., [29]. The depth measurements of the Microsoft Kinect are derived from the disparities, which are normalized and quantized as 11-bit integers. The effect of disparity measurement quantization on the depth measurement precision, which degrades with the square of the depth, is discussed by Chow and Lichti [29] and is analyzed in detail herein. Asad and Abhayaratne [21] present a method with morphological filtering to reduce the quantization error. A straightforward method to reduce quantization error by spatial averaging many depth measurements is proposed herein.

## 3. Deflection Measurement Methods

### 3.1. Three-Dimensional Coordinates from Microsoft Kinect

According to Khoshelham and Elberink [27], 3D coordinates can be derived from the Microsoft Kinect depth data as follows:

(1) $$\{\begin{array}{c} X_{k}=-\frac{{\text{depth}}_{k}}{f}(x_{k}-x_{p}+\Delta x)\\ Y_{k}=-\frac{{\text{depth}}_{k}}{f}(y_{k}-y_{p}+\Delta y)\\ Z_{k}={\text{depth}}_{k} \end{array}$$

where (*x_k_, y_k_*) are the image coordinates of the depth image point *k*; (*x_p_, y_p_*) are the coordinates of the principal point; (Δ*x*, Δ*y*) are correction terms for lens distortions; (*X_k_, Y_k_, Z_k_*) are the 3D coordinates of the object point *k; f* is focal length of the IR depth camera and *depth_k_* is the depth measurement.

### 3.2. Mathematical Model for Concrete Beam Deflection

Often in fatigue testing the load is applied at a single frequency with a sinusoidal displacement waveform. The measurement objective is to automatically reconstruct the resulting sinusoidal displacement of the loaded beam from sensor data. A time series of depth measurements captured with a Kinect can be used to reconstruct this displacement signal. The displacement can be modeled as follows:

(2) $$h\left(t\right)=C\cos2\pi f_{0}t+D\text{sin}2\pi f_{0}t+E$$

where *f*_0_ is the loading frequency, *C* and *D* are the coefficients of the harmonic model and, *E* is the mean value of the time series.

**Figure 2.**
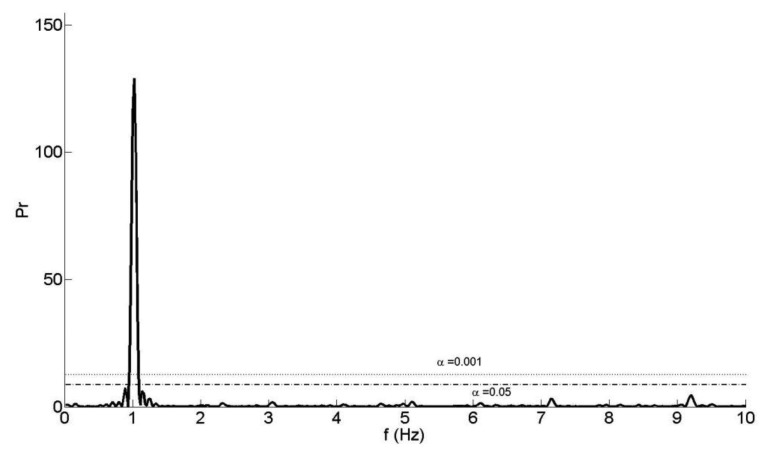
PSD of the depth time series.

The first step of our reconstruction algorithm is the initial approximation of some displacement parameters from the spectrum of the depth measurement time series. Although the frame sampling rate of the Microsoft Kinect is nominally uniform, random drop-outs can occur due to the USB data transmission. To overcome the missing data problem, Lomb's method [30] is used to calculate the power spectral density (PSD) from the time series. Then, the nominal loading frequency, *f*_0_ of the sinusoidal motion is identified from the dominant peak (Figure 2).

The false-alarm probability of the time-series, *α*, is calculated to confirm the presence (or the absence) of a periodic signal and to assess the significance of the dominant peak in the PSD. A small value for the false-alarm probability indicates a highly significant periodic signal. If *α* is less than 0.001, a highly significant periodic signal exists in the time series and, if *α* is greater than 0.05, then the signal is noise [30].

With the approximate loading frequency treated as a constant, *f*_0_, the coefficients *C, D, E* are estimated by least-squares under the assumption of a linear functional model. In the final step all four coefficients of the non-linear model are simultaneously estimated by least-squares using the approximate coefficients as initial values for the Taylor series expansion [31]. The amplitude *A* of the motion, one of the key parameters for the structural analysis, is then derived as:

(3) $$A=\sqrt{C^{2}+D^{2}}$$

## 4. Experiment and Description

### 4.1. Experiment Design and Data Acquisition

**Figure 3.**
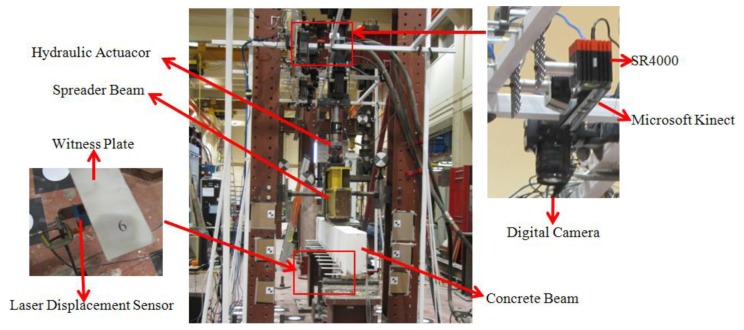
Photographic image of the experiment setup.

A 3.3 m long concrete beam with 150 mm × 300 mm rectangular cross-section was supported over a 3 m long span (Figure 3) for the fatigue loading test. The beam was reinforced internally with steel bars and stirrups and externally with a steel fibre reinforced polymer (SRP) sheet bonded to the beam soffit over the entire span. The SRP sheet was used for the purpose of a separate investigation into its efficiency in flexural strengthening of reinforced concrete elements for fatigue resistance. A hydraulic actuator was used to apply a periodically-varying load at two points, each 300 mm on either side of the concrete beam's mid span, via a 1,400 mm long steel spreader beam.

The experiment comprised two static loading and fatigue loading cycles. The periodic loading cycles—of interest here—were applied to the concrete beam based on load control. First, 36,000 load cycles were applied from 24 kN to 72 kN at 3 Hz loading frequency causing 2.6 mm displacement amplitude at the mid-span of the beam. In general, fatigue loading tests are conducted at 3 Hz [17,18]. However, in order to meet the sampling frequency requirement of the digital camera system [7], load testing was also conducted at 1 Hz.

Ideally the surface of the concrete beam would be directly measured with the optical sensors. However, nearly 50% of its top surface at mid-span was occluded by the spreader beam. Thus a targeting means was required to facilitate optical measurement of the concrete beam. As described in [20], the target system comprised thirteen white-washed, thin aluminum witness plates (220 mm × 50 mm) bonded to the side of the beam at an interval of 250 mm along its length and numbered 1 to 13 (see Figure 3). The witness plate dimensions were chosen in such a way that the plate would not affect the concrete beam rigidity and hence its deflection. Wider plates, which would be advantageous from an imaging perspective, were ruled out as they would also interact with the cracks in the beam, delaying their formation and restraining their widths. The end result would be a stiffer beam with less deflection.

The measurement systems used for the experiment included one Microsoft Kinect, three SR4000 time-of-flight range cameras, a photogrammetric system comprised a set of eight digital cameras and two projectors, and five LDSs. Only the Microsoft Kinect results are presented and analyzed here. Qi and Lichti [20] report the results of the deflection measurements obtained with the SR4000 range cameras while [16] present the photogrammetric system results.

All sensors were mounted on a rigid scaffold assembly approximately 1.9 m above the top surface of the concrete beam. At this depth the Microsoft Kinect depth precision, which varies inversely with depth squared, is just under 10 mm [32]. The Microsoft Kinect was warmed up for one hour prior to the fatigue loading test in order to obtain stable measurement data. Chow *et al.* [33] recommend that at least one hour warm-up is necessary to obtain stable depth measurements. Since only relative displacement measurements were required and the beam displacements were small (8 mm peak-to-peak), the geometric calibration of the Kinect was not required. As shown in [19], subtraction of acquired depth measurements from a zero-load reference has the effect of removing any biases due to un-modeled systematic errors. Many 5 s long Kinect datasets were collected at the 30 Hz acquisition rate throughout the testing regime. For each loading frequency, five datasets were analyzed, each one being randomly selected from one of the five days of load testing. The elapsed time between the first and last 1 Hz and 3 Hz datasets was 55 min and 2 h 28 min, respectively.

The accuracy of the Microsoft Kinect was assessed by comparison with the measurements from KEYENCE LKG407 CCD LDSs. The manufacturer stated linearity of this sensor is 0.05% of the 100 mm measurement range and the precision is 2 μm. Five LDSs were placed under the centroid of thin plates along the length of the beam. Data were acquired with these active triangulation systems at 300 Hz at the same time as the Kinect to permit direct comparison of the reconstructed displacements.

### 4.2. Depth Data Extraction

**Figure 4.**
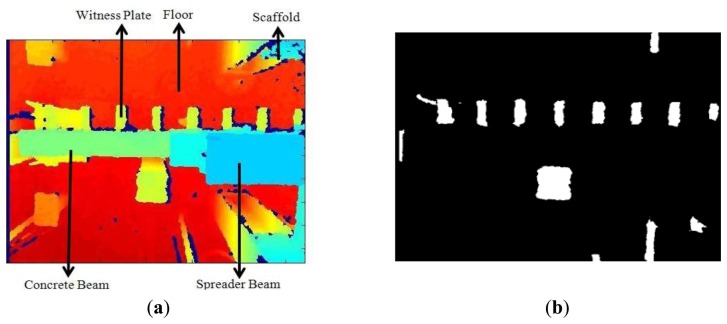

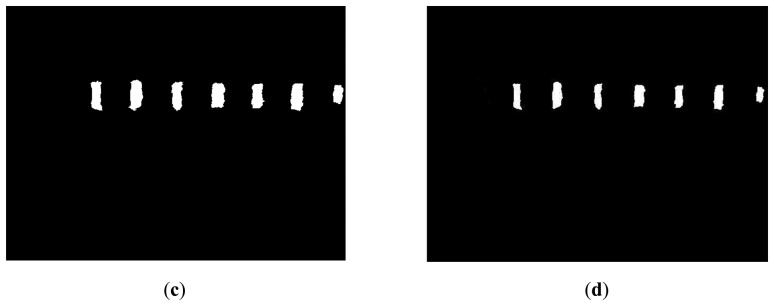
(**a**) Depth image from Microsoft Kinect, (**b**) The binary image after depth-based segmentation, (**c**) The binary image after eccentricity analysis, and (**d**) The final result after image erosion.

Although the Microsoft Kinect provides both depth and RGB images, only the depth image (Figure 4a) was used to extract the witness plates in order to overcome the obstacle of the different fields-of-view of the RGB and IR cameras. For each image in an acquired time series, depth-based segmentation [20] was performed to remove the floor and objects above the witness plates. The resulting binary image is shown in Figure 4b. Second, the eccentricity [34] of the connected regions in the binary image was used to distinguish the thin plates (Figure 4c) from unwanted regions. One witness plate at the end of the beam was excluded by the algorithm due to segmentation errors caused by depth measurements from the beam support. This was not a problem in the ensuing structural analysis since there was little or no motion at the end of the beam. In the last step, image erosion was used to remove the edges of the witness plates (Figure 4d). The 3D centroid of each witness plate region was then calculated and used for the displacement signal reconstruction. Any differences in vertical displacement between the edges of the witness plates are eliminated by the spatial averaging operation.

## 5. Results and Analysis

### 5.1. Quantization Error Analysis

**Figure 5.**
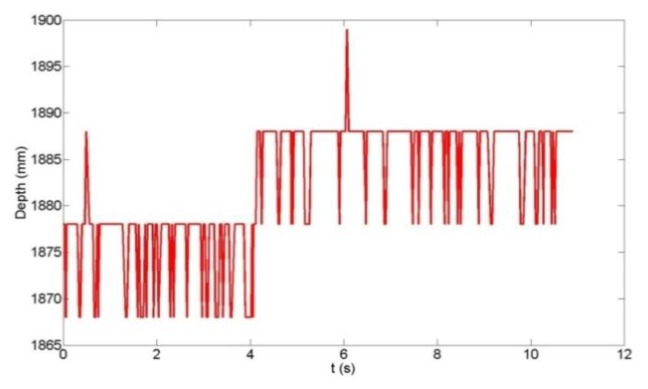
Raw Microsoft Kinect depth measurement at the witness plate centroid.

**Figure 6.**
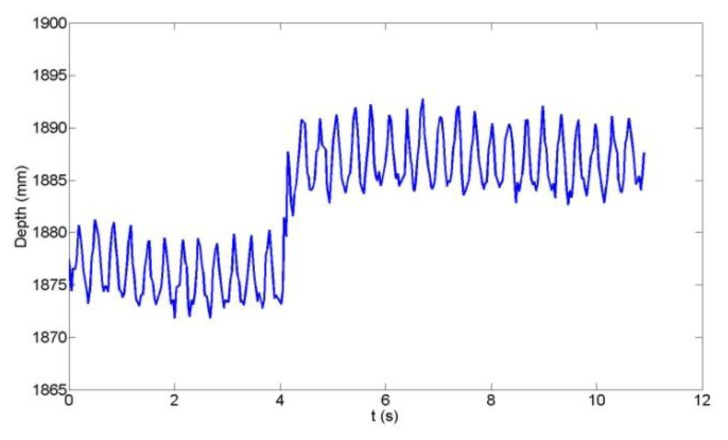
Computed witness plate centroid depth measurement.

Since the depth measurement accuracy is strongly influenced by the Microsoft Kinect's inherent disparity quantization [27], an analysis of the quantization error effects on the vertical deflection reconstruction was conducted. Figure 5 shows the time-series of the raw depth measurement of a witness plate at the centroid pixel location, while Figure 6 shows the time series of the computed centroid depth using the algorithm described in Section 4.2. The quantization error effects are clearly visible in Figure 5 as the 10 mm steps in depth and the sinusoidal witness plate motion cannot be inferred from the time series. In Figure 6, however, the effect of the spatial averaging of depth values over the witness plate region is evident as the sinusoidal motion (∼6 mm peak-to-peak displacement in this example) is visible. High-precision displacement estimates can be expected since the spatial averaging reduces the theoretical quantization error standard deviation [35] as follows:

(4) $$\sigma_{z}=\frac{\Delta }{\sqrt{12}\sqrt{N}}$$

where Δ is the quantization step in depth and *N* is the number of spatially-averaged depth samples. In this example with Δ = 10 mm, the depth precision is improved from 2.89 mm (*N* = 1) to 0.11 mm (*N* = 700) by averaging the depth measurements within the witness plate region. Even with the spatial averaging, a 10 mm discontinuity still exists at 4.1 s. This can be easily identified by differentiating the time series and locating the peak. Only data between such discontinuities are then utilized in the displacement signal reconstruction. The luxury of this technique is afforded by the fact that the quantization step is larger than the beam displacement. Though use of a larger plate would provide more samples for the spatial averaging and improve data quality even further, this was not possible for the reasons discussed in Section 4.1.

### 5.2. Reconstructed Concrete Beam Deflection Results

**Figure 7.**
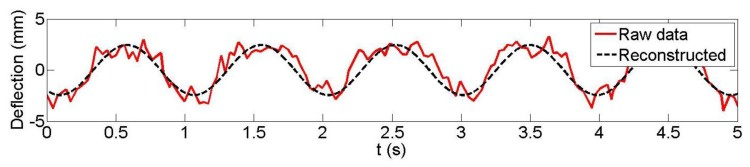
Kinect observed and reconstructed vertical displacements (*f*_0_ = 1 Hz), dataset 4 witness plate 7.

**Figure 8.**
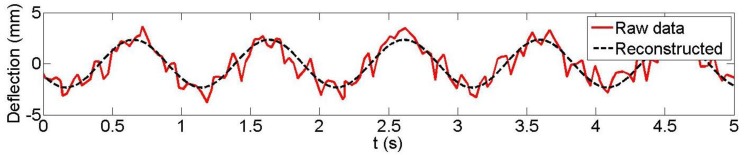
Kinect observed and reconstructed vertical displacements (*f*_0_ = 1 Hz), dataset 5 witness plate 7.

Following the previously-outlined procedure, the results achieved for the reconstruction of the vertical displacement of three plates (3, 5 and 7; plate 7 was located at the mid-span of the concrete beam; plate 3 was near the end) from the Microsoft Kinect data are analyzed for both 1 Hz and 3 Hz loading frequencies. LDS data were available at these three plates. Figures 7 and 8 are typical examples of the observed and reconstructed witness plate centroid trajectories with a 1 Hz nominal loading frequency. Table 1 presents the recovered amplitudes and loading frequencies derived from Microsoft Kinect depth data measurement for the five different datasets, their estimated differences with the LDS (accuracy measures ΔA and Δ*f*_0_) and the estimated precision measures. As can be seen, sub-millimetre amplitude precision and accuracy were achieved in all but one case (dataset 2, plate 5). The estimated loading frequency precision and absolute accuracy were on the order of a few mHz with only two exceptions.

**Figure 9.**
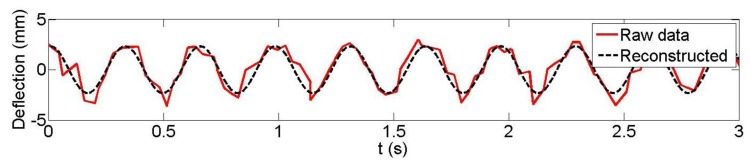
Kinect observed and reconstructed vertical displacements (*f*_0_ = 3 Hz), dataset 2 witness plate 7.

**Figure 10.**
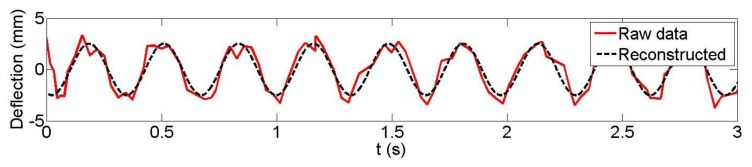
Kinect observed and reconstructed vertical displacements (*f*_0_ = 3 Hz), dataset 5 witness plate 7.

Figures 9 and 10 depict the observed and reconstructed witness plate centroid trajectories with 3 Hz nominal loading frequency. Table 2 presents the recovered loading frequencies and amplitudes derived from the Microsoft Kinect data, their estimated differences with the LDS and the estimated precision measures. The amplitude precisions and differences are of similar magnitudes to those of the 1 Hz loading frequency results. Additionally, the loading frequency differences are on the order of a few mHz or less and with only one exception in this case.

### 5.3. Discussion

The best-case displacement amplitude accuracy achieved was 0.05 mm and, although the worst-case amplitude accuracy of −1.63 mm represents a significant proportion of the displacement amplitude, the results are much improved over the previously-reported centimetre-level depth precision [32]. The few inaccurate amplitudes and frequencies can be attributed to residual quantization errors. The results demonstrate that small displacement measurements can be made with sub-millimetre accuracy with the Microsoft Kinect by using straightforward spatial filtering and modelling techniques. However, they are not as accurate as what can be achieved with the SR4000 time-of-flight camera [20]. The results presented are independent of the loading frequency, though only two (1 Hz and 3 Hz) were tested and they are well below the 15 Hz Nyquist frequency of the Microsoft Kinect. The missing data problem was only a minor obstacle for the initial stage of the signal reconstruction; it was not an issue for the final signal reconstruction since uniformly-sampled data were not required.

## 6. Conclusions and Future Work

The Microsoft Kinect has been successfully used to measure the periodic vertical displacement of a concrete beam during cyclic loading tests. Automated algorithms for witness plate extraction, spatial data filtering and signal reconstruction were developed for the experiments. The principal advantages of the Microsoft Kinect for structural measurements include: the sensor's wide field-of-view (allowing imaging of a large area—half of the concrete beam—unlike the more accurate LDS point measurement device); the use of straightforward filtering and modelling techniques that allowed sub-millimetre displacements to be measured from a sensor for which the nominal accuracy is 10 mm at the 2 m stand-off distance of the experiments.

In this paper only vertical displacements—which were of primary interest—were reported. Future work will concentrate on the extraction of three-dimensional displacement data, which can have value for assessing load eccentricity. This will require improvements to the segmentation algorithm in order to produce more accurate witness plate regions. Cues derived from the RGB imagery will likely be beneficial to this process, but this will require proper registration with the depth imagery after calibration [29]. Further work is also needed to overcome the residual quantization errors that were encountered in a couple of the datasets. Future work should also concentrate on more general, multi-frequency loading conditions since the case of only a single unknown loading frequency has been investigated here.

## Figures and Tables

**Table 1.** Recovered loading frequencies and amplitudes for Plates 3, 5 and 7 (*f*_0_ = 1 Hz). *A_KIN_*, *f*_0−_*_KIN_*, and *σ_A_*_−_*_KIN_*, *σ*_*f*_0−*KIN*__ and *σ_r̂_*_−_*_KIN_* are the amplitude, loading frequency and their corresponding standard deviations estimated from the Kinect data; *A_LDS_*, *f*_0−_*_LDS_*, *σ_A_*_−_*_LDS_* and *σ*_*f*_0−*LDS*__ are the amplitude, loading frequency and their corresponding standard deviations estimated from the LDS data.

**Plate #**	**Set #**	***A_KIN_*** **(mm)**	***A_LDS_*** **(mm)**	***f*_0−_*_KIN_*** **(Hz)**	***f*_0−_*_LDS_*** **(Hz)**	**Δ*A*** **(mm)**	**Δ*f*_0_** **(mm)**	***σ_A_*_−_*_KIN_*** **(mm)**	***σ*_*f*_0−*KIN*__** **(mm)**	***σ_r̂_*_−_*_KIN_*** **(mm)**
3	1	0.72	1.12	0.9125	1.0222	−0.40	−0.1097	0.17	0.0320	1.26
	2	1.34	1.20	1.0192	1.0223	0.14	−0.0031	0.09	0.0123	0.59
	3	1.67	1.22	1.0276	1.0224	0.45	0.0052	0.07	0.0040	0.67
	4	1.54	1.21	1.0239	1.0222	0.33	0.0017	0.05	0.0031	0.48
	5	1.99	1.21	1.0231	1.0222	0.78	0.0009	0.05	0.0012	0.63

5	1	2.11	2.22	1.0240	1.0222	−0.11	0.0018	0.12	0.0083	0.95
	2	0.65	2.28	1.0039	1.0222	−1.63	−0.0183	0.09	0.0272	0.62
	3	1.51	2.28	1.0266	1.0224	−0.77	0.0042	0.11	0.0148	0.76
	4	2.23	2.30	1.0252	1.0222	−0.07	0.0030	0.08	0.0036	0.75
	5	1.60	2.30	1.0220	1.0222	−0.70	−0.0002	0.05	0.0018	0.69

7	1	3.02	2.54	1.0311	1.0222	0.48	0.0089	0.13	0.0057	1.51
	2	2.66	2.57	1.0207	1.0223	0.09	−0.0016	0.11	0.0036	0.67
	3	2.48	2.59	1.0284	1.0224	−0.11	0.0060	0.10	0.0041	0.91
	4	3.36	2.61	1.0214	1.0222	0.75	−0.0008	0.08	0.0012	1.12
	5	2.33	2.61	1.0209	1.0222	−0.28	−0.0013	0.08	0.0017	0.83

**Table 2.** Recovered loading frequencies and amplitudes for Plates 3, 5 and 7 (*f*_0_ = 3 Hz). *A_KIN_*, *f*_0−_*_KIN_*, *σ_A_*_−_*_KIN_*, *σ*_*f*_0−*KIN*__ and *σ_r̂_*_−_*_KIN_* are the amplitude, loading frequency and their corresponding standard deviations estimated from the Kinect data; *A_LDS_*, *f*_0−_*_LDS_*, *σ_A_*_−_*_LDS_* and *σ*_*f*_0−*LDS*__ are the amplitude, loading frequency and their corresponding standard deviations estimated from the LDS data.

**Plate #**	**Set #**	***A_KIN_*** **(mm)**	***A_LDS_*** **(mm)**	***f*_0−_*_KIN_*** **(Hz)**	***f*_0−_*_LDS_*** **(Hz)**	**Δ*A*** **(mm)**	**Δ*f*_0_** **(mm)**	***σ_A_*_−_*_KIN_*** **(mm)**	***σ*_*f*_0−*KIN*__** **(mm)**	***σ_r̂_*_−_*_KIN_*** **(mm)**
3	1	1.45	1.22	3.0653	3.0686	0.23	−0.0033	0.06	0.0039	0.57
	2	0.77	1.20	3.0670	3.0680	−0.43	−0.0010	0.08	0.0183	0.51
	3	1.94	1.20	3.0735	3.0680	0.74	0.0055	0.11	0.0098	0.81
	4	1.49	1.20	3.0666	3.0675	0.29	−0.0009	0.04	0.0011	0.67
	5	1.29	1.27	3.0597	3.0675	0.02	−0.0078	0.06	0.0066	0.48

5	1	1.56	2.31	3.0565	3.0686	−0.75	−0.0121	0.07	0.0080	0.45
	2	1.32	2.33	3.0467	3.0675	−1.01	−0.0208	0.17	0.0237	1.12
	3	1.60	2.33	3.0926	3.0675	−0.73	0.0251	0.10	0.0102	0.70
	4	2.13	2.33	3.0666	3.0675	−0.20	−0.0009	0.05	0.0009	0.83
	5	2.29	2.34	3.0590	3.0675	−0.05	−0.0085	0.12	0.0072	0.94

7	1	2.18	2.54	3.0709	3.0686	−0.36	0.0023	0.13	0.0057	0.63
	2	2.33	2.62	3.0665	3.0679	−0.29	−0.0014	0.10	0.0045	0.82
	3	2.51	2.62	3.0766	3.0680	−0.11	0.0086	0.14	0.0094	1.05
	4	2.04	2.65	3.0663	3.0675	−0.61	−0.0012	0.06	0.0008	0.70
	5	2.81	2.64	3.0660	3.0675	0.17	−0.0015	0.09	0.0022	1.13
